# Analysis of Gene-Environment Interactions Related to Developmental Disorders

**DOI:** 10.3389/fphar.2022.863664

**Published:** 2022-03-17

**Authors:** Yuhei Nishimura, Kenji Kurosawa

**Affiliations:** ^1^ Department of Integrative Pharmacology, Mie University Graduate School of Medicine, Tsu, Japan; ^2^ Division of Medical Genetics, Kanagawa Children’s Medical Center, Yokohama, Japan; ^3^ Department of Clinical Dysmorphology, Mie University Graduate School of Medicine, Tsu, Japan

**Keywords:** developmental toxicity, gene-environment interaction, susceptibility, resilience, organoid, clinical genetics

## Abstract

Various genetic and environmental factors are associated with developmental disorders (DDs). It has been suggested that interaction between genetic and environmental factors (G × E) is involved in the etiology of DDs. There are two major approaches to analyze the interaction: genome-wide and candidate gene-based approaches. In this mini-review, we demonstrate how these approaches can be applied to reveal the G × E related to DDs focusing on zebrafish and mouse models. We also discuss novel approaches to analyze the G × E associated with DDs.

## Introduction

**FIGURE 1:**
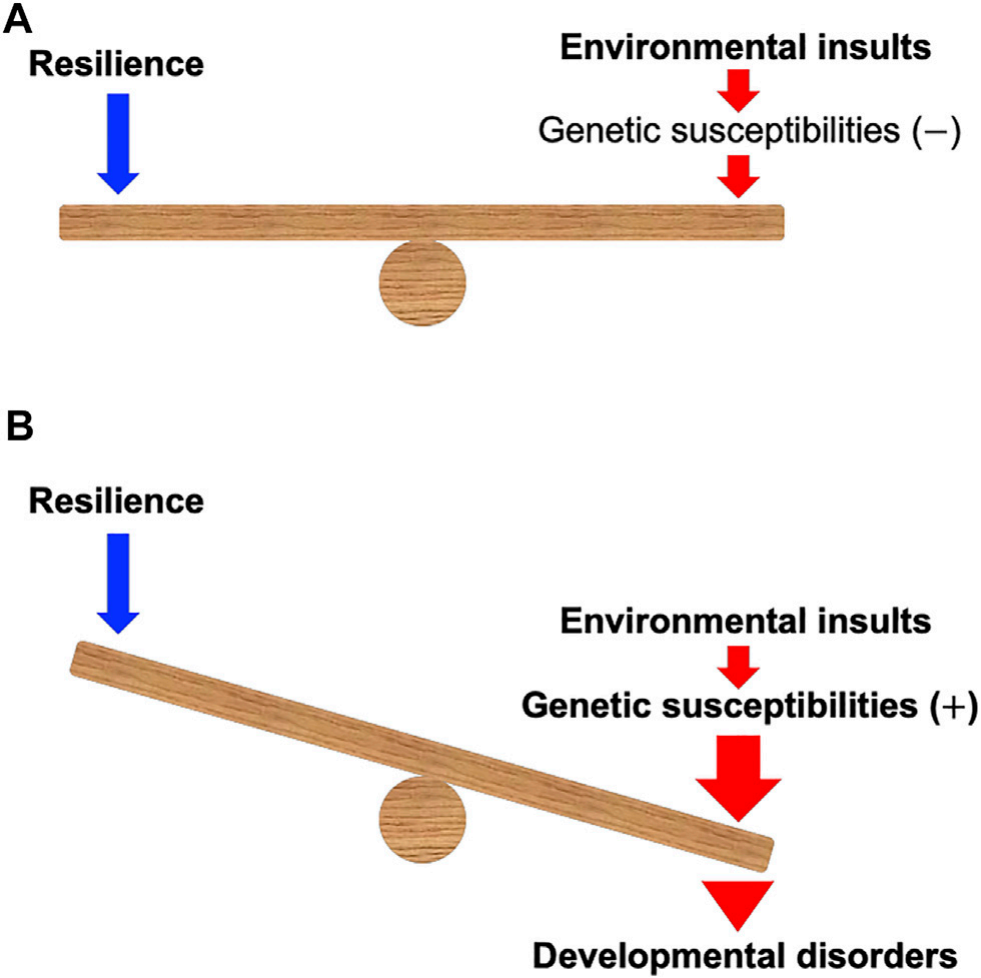
A model of gene–environment interaction proposed in this mini-review. **(A)** When environmental insults during development can be counterbalanced by the resilience in the individuals without the genetic susceptibilities, the insults may not cause developmental disorders. **(B)** Environmental insults during development can be enhanced and defeat the resilience of the individuals with the genetic susceptibilities, which may cause developmental disorders. Note that genetic susceptibilities may modulate the power of resilience.

Developmental toxicity linked to early-life chemical exposure can have a crucial impact on the development of various tissues and is associated with developmental disorders (DDs) such as fetal alcohol syndrome (FAS), autism spectrum disorder (ASD), attention deficit hyperactivity disorder (ADHD), craniofacial anomalies, and congenital heart defects (De la Monte and Kril, 2014; Bölte et al., 2019; Beames and Lipinski, 2020; Hollander et al., 2020; Kalisch-Smith et al., 2020; Martinelli et al., 2020). The susceptibility to these chemicals may be determined by genetic factors (Lovely et al., 2017; Musci et al., 2019; Beames and Lipinski, 2020; Boyce et al., 2020; Gomes et al., 2021) (Figure 1). The gene**–**environment interaction (G × E) may affect the balance between resilience and the risk of DDs (Cicchetti and Rogosch, 2012; Elbau et al., 2019; Molnar-Szakacs et al., 2021).

G × E can be analyzed using genome-wide and candidate gene-based approaches (Elbau et al., 2019; Gomes et al., 2021). For example, analysis of samples using the Simons Simplex Collection (Fischbach and Lord, 2010) combined with array comparative genomic hybridization screening revealed the interactive effects of copy number variations (CNV) and maternal infection on the risk of ASD (Mazina et al., 2015). A genome-wide approach using the Simons Simplex Collection also revealed the interactive effects of prenatal antidepressant exposure and the corresponding gene mutations on the severity of ASD (Ackerman et al., 2017). A population-based case-control study found that the joint effect of CNV and air pollution exposure increased the risk of ASD (Kim et al., 2017). Candidate gene approaches demonstrated that the interactions between maternal genotype of paraoxonase 1, a key enzyme in the metabolism of organophosphates, and prenatal exposure to organophosphates impacted cognitive development in the child (Engel et al., 2011), and also that interaction between a functional promoter variant in the MET receptor tyrosine kinase gene of children and air pollution exposure increased the risk of ASD (Volk et al., 2014). However, studying G × E in the human population is still challenging because of various reasons, including the difficulties in selecting genetic variants, the study design, the environmental factors of interest, and the temporality of environmental exposure (Mcallister et al., 2017; Esposito et al., 2018). Animal models have been successfully used to analyze G × E and its impact on developmental defects (Eberhart and Parnell, 2016; Hong and Krauss, 2018; Beames and Lipinski, 2020; Lovely, 2020; Raterman et al., 2020; Fernandes and Lovely, 2021). We review G × E studies applying genome-wide and candidate gene-based approaches using zebrafish and mouse models, especially focusing on gene-ethanol interaction, and their impact on developmental defects (Table 1).

**TABLE 1:** The reviewed studies of gene–environment interactions related to developmental disorders.

Exposure	App, sp	Genetic susceptibilities	Phenotypic outcomes	References
Ethanol	GW, Dr	Mutation in *si:dkey-88l16.3*	Craniofacial anomalies	Swartz et al. (2020)
Ethanol	CG, Dr	Knockdown or haploinsufficiency of *shh*	Craniofacial anomalies	Zhang et al. (2011); Everson et al. (2020)
Ethanol	CG, Mm	haploinsufficiency of *Shh* or *Gli2*	Craniofacial anomalies	Kietzman et al. (2014)
Ethanol	CG, Mm	Mutation in *Cdon*	Holoprosencephaly	Hong and Krauss, (2012)
Ethanol	CG, Dr	Haploinsufficiency of *vangl2*	Craniofacial anomalies	Swartz et al. (2014)
Ethanol	CG, Dr	Mutation in *pdgfra*	Craniofacial anomalies	Mccarthy et al. (2013)
Abamectin	GW, Dr	Mutation in *sox7* promoter	Craniofacial anomalies, pericardial edema, scoliosis	Balik-Meisner et al. (2018)
PBO	CG, Dr	Haploinsufficiency of *shh*	Craniofacial anomalies	Everson et al. (2020)
PBO	CG, Dr	Haploinsufficiency of S*hh*	Holoprosencephaly	Everson et al. (2019)
Vismodegib	CG, Mm	Haploinsufficiency of *Gli2*	Holoprosencephaly	Heyne et al. (2016)
Blebbistatin	CG, Dr	Haploinsufficiency of *vangl2*	Craniofacial anomalies	Sidik et al. (2021)

PBO, piperonyl butoxide; Ap, Approach; Sp, species; GW, genome-wide approach; CG, candidate gene-based approach; Dr, *danio rerio*; Mm, *mus musculus*.

### Genetic Susceptibilities to Developmental Ethanol Exposure

#### Genome-Wide Approaches

Zebrafish have been successfully used to identify the genes involved in disease development through unbiased forward genetic screening with chemical mutagenesis (Mullins et al., 1994; Kelsh et al., 1996; Amsterdam and Hopkins, 2006; Swartz et al., 2020). The signaling pathways involved in cranial neural crest development are impaired in FAS, leading to various craniofacial anomalies such as cleft palate and holoprosencephaly (Smith et al., 2014; Nasreddine et al., 2021). To examine this, an N-ethyl-N-nitrosourea (ENU)-based random mutagenesis was performed to identify novel ethanol-sensitive zebrafish mutants, wherein F3 embryos from 126 inbred F2 families were exposed to 1% ethanol in the medium from 6 h post-fertilization (hpf) until they were screened. Alcian Blue/Alizarin Red staining was performed 4–7 days post-fertilization (dpf) to examine alterations in the craniofacial skeleton. The screening identified a novel ethanol-sensitive mutant in which the splice donor of exon 15 in *si:dkey-88l16.3*, a previously uncharacterized gene, was mutated (Swartz et al., 2020). The mechanisms of how the impairment of si:dkey-88l16.3 is involved in the craniofacial defects remain to be clarified.

#### Candidate Gene-Based Approaches

Genes involved in sonic hedgehog (SHH) signaling pathways have been intensively analyzed in studies on G × E associated with FAS. In zebrafish, knockdown or haploinsufficiency of *shh* sensitizes embryos to alcohol-induced craniofacial defects (Zhang et al., 2011; Everson et al., 2020). In mice, haploinsufficiency of *Shh*, or *Gli2*, which encodes a zinc finger transcription factor that acts as a mediator of hedgehog signaling, increases sensitivity to ethanol-induced holoprosencephaly (Kietzman et al., 2014).

A screen of zebrafish mutants found that VANGL planar cell polarity protein 2 (vangl2) is involved in the genetic susceptibility to craniofacial defects induced by developmental ethanol exposure (Swartz et al., 2014). VANGL2 is a transmembrane protein that regulates the Wnt-mediated planar cell polarity (PCP) pathway (Yang and Mlodzik, 2015; Bailly et al., 2018; Bell et al., 2021). Zebrafish with mutations of *vangl2* show slightly shortened craniofacial elements when there is no exposure to ethanol, whereas severe craniofacial anomalies such as synophthalmia, rod-like ethmoid plate, and disrupted axon projections, are observed in the *vangl2* mutant exposed to ethanol during development (Swartz et al., 2014).

Impairment of platelet-derived growth factor (PDGF) receptor α (PDGFRA) and the resultant mutation in the 3′ untranslated region (UTR) of the *PDGFRA* gene (c.*34G > A) is associated with cleft palate in humans, mice, and zebrafish (Xu et al., 2005; Eberhart et al., 2008; Rattanasopha et al., 2012). MicroRNA (miRNA) 140 (miR-140) binds to the 3′-UTR of *pdgfra* and suppresses the expression of Pdgfra in zebrafish (Eberhart et al., 2008). The suppression of PDGFRA by miR-140 is also observed in cultured mouse palate cells (Li et al., 2019a). The c.*34G > A mutation is located 10 bp away from a predicted binding site of miR-140 (Rattanasopha et al., 2012). Ethanol exposure increases miR-140 levels in the extracellular vesicles of fetal neural stem cells (Tseng et al., 2019). Pdgfra is protective against ethanol-induced craniofacial anomalies in zebrafish (Mccarthy et al., 2013). These findings suggest that miR-140-mediated PDGFRA expression may be involved in the susceptibility to ethanol that is associated with craniofacial anomalies.

### Genetic Susceptibilities to Other Developmental Toxicants

#### Genome-Wide Approaches

Genetic diversity in zebrafish populations can be used to analyze G × E. A large-scale drug screening for the assessment of developmental toxicity in a zebrafish line found that abamectin, a widely used insecticide and anthelmintic, elicited differential responses in the population (Balik-Meisner et al., 2018). A genome-wide association study (GWAS) using 276 individual zebrafish, either susceptible or resistant to the developmental toxicity of abamectin, identified a G/T variant in the promoter region of sox7 to be associated with this differential response in the population (Balik-Meisner et al., 2018). The T allele frequency of affected and unaffected individuals was 45 and 12%, respectively, and the expression of sox7 after abamectin exposure in affected individuals was significantly lower than that in unaffected individuals (Balik-Meisner et al., 2018). Ablation of Sox7 and mutation of sox7 in mice and zebrafish, respectively, can cause pericardial edema, which is a phenotype observed in the developmental toxicity of abamectin (Wat et al., 2012; Hermkens et al., 2015). These findings suggest that the single nucleotide variation (SNV) at the sox7 promoter is involved in susceptibility to the developmental toxicity of abamectin. However, the possibility that polygenic functions are involved in the differential susceptibility cannot be excluded (Balik-Meisner et al., 2018). Rodent population models such as the Hybrid Mouse Diversity Panel, Collaborative Cross, and Diversity Outbred, have been successfully used to identify novel genes that are susceptible to environmental exposure (Harrill and Mcallister, 2017). Various other methodologies to analyze G × E have also been actively developed (Mcallister et al., 2017; Esposito et al., 2018). Multiple gene functions that are related to the susceptibility to environmental exposure and are causative of DDs may be elucidated by utilizing these new methodologies.

#### Candidate Gene-Based Approaches

Cranial neural crest cells regulate craniofacial development through multiple pathways, including SHH, and Wnt/PCP signaling pathways (Bush and Jiang, 2012; Suzuki et al., 2016). Candidate gene-based approaches that target genes involved in these pathways have successfully identified that G × E is associated with craniofacial anomalies such as holoprosencephaly and cleft palate (Eberhart and Parnell, 2016; Hong and Krauss, 2018; Beames and Lipinski, 2020; Lovely, 2020; Raterman et al., 2020; Fernandes and Lovely, 2021).

There are global chemicals and therapeutic drugs that can affect SHH signaling. For example, piperonyl butoxide (PBO), a semisynthetic pesticide synergist present in hundreds of commercial products, can inhibit SHH signaling (Wang et al., 2012). In mice and zebrafish with haploinsufficiency of SHH, the embryos are sensitized to craniofacial defects induced by PBO (Everson et al., 2019; Everson et al., 2020).

Cholesterol is required in the SHH signaling cascade (Haas and Muenke, 2010). Statins, therapeutic drugs for hypercholesterolemia, negatively affect SHH signaling through the inhibition of 3-hydroxy-3-methyl-glutaryl-CoA reductase (HMGCR) that is a key enzyme in cholesterol synthesis (Haas and Muenke, 2010; Abramyan, 2019). In zebrafish, orofacial defects are induced by the developmental exposure to statins or mutation of *hmgcr* (Signore et al., 2016). Mutation of 7-dehydrocholesterol reductase gene that encodes an enzyme involved in cholesterol metabolism, is the cause of Smith-Lemli-Opitz syndrome (SLOS), a DD with multiple congenital anomalies including cleft palate and holoprosencephaly (Kelley and Hennekam, 2000). The severity of SLOS depends on the maternal apo E genotype (Witsch-Baumgartner et al., 2004). Apo E, a protein regulating the transport of cholesterol and other lipids in the blood and the brain, includes three common isoforms: ApoE2, ApoE3, and ApoE4 (Villeneuve et al., 2014). Because ApoE2 is defective in binding to low-density lipoprotein receptors, plasma total cholesterol level tends to be low in individuals with the ApoE2 genotype (Witsch-Baumgartner et al., 2004). The severity score of SLOS is higher in children from mothers with the ApoE2 genotype than from those without it (Witsch-Baumgartner et al., 2004). Individuals with the ApoE2 genotype are more sensitive to statin therapy than those with the ApoE4 genotype (Mega et al., 2009). A link between cholesterol metabolism and ASD has been suggested (Gillberg et al., 2017). These studies suggest that G × E involved in cholesterol metabolism may be associated with DDs.

Mice with single-allele *Gli2* mutation show an increased incidence of holoprosencephaly induced by vismodegib, a hedgehog pathway inhibitor (Heyne et al., 2016). Mice with a null mutation of *Cdon*, which encodes an SHH co-receptor, are sensitized to prenatal ethanol exposure to produce holoprosencephaly with defective expression of genes targeted by SHH (Hong and Krauss, 2012). Apart from the susceptibility to these teratogens, mice with haploinsufficiency of *Shh* or *Gli2*, or null allele of *Cdon*, are phenotypically indistinguishable from the wild-type littermates (Hong and Krauss, 2012; Kietzman et al., 2014; Heyne et al., 2016). In contrast, mice with a null mutation of *Mosmo*, which encodes a component of a membrane protein complex called MMM that promotes degradation of the Hedgehog signal transducer Smoothened, show multiple birth defects with increased SHH signaling (Kong et al., 2021). These birth defects can be suppressed by *in utero* treatment with vismodegib to inhibit SHH signaling (Kong et al., 2021). These studies suggest that individuals with mutations involved in the SHH pathway may be susceptible to chemicals that affect SHH signaling.

Mutation of *vangl2* sensitizes the zebrafish to craniofacial anomalies induced by blebbistatin, an inhibitor of the Wnt/PCP pathway (Sidik et al., 2021). Genes involved in the PCP pathway have also emerged as susceptibility-inducing genes in ASD and other DDs (Sans et al., 2016; Milgrom-Hoffman and Humbert, 2018). Therefore, G × E affecting PCP pathways warrants further investigation.

Non-coding RNA such as miRNA and long non-coding RNA (lncRNA) have also attracted attention as important mediators in response to environmental stressors (Miguel et al., 2020). For example, lncRNA is involved in the toxic response to dioxins, such as jaw malformation and pericardiac edema, by downregulating the expression of sox9b (Mathew et al., 2008; Xiong et al., 2008; Garcia et al., 2018). The roles of non-coding RNA in G × E associated with ASD have been actively studied (Beversdorf et al., 2021; Cui et al., 2021).

## Discussion

Advances in genome editing technologies have enabled us to edit any gene of interest in various experimental models, including zebrafish and induced pluripotent stem (iPS) cells generated from human samples (Adachi et al., 2022; Whiteley et al., 2022). Public databases focusing on genes, biological samples, and chemicals related to various diseases including DDs, have been expanding (Al-Jawahiri and Milne, 2017; Reilly et al., 2017; Lombardo et al., 2019; Davis et al., 2021). These resources have accelerated G × E-focused research related to DDs.

Brain organoids derived from human iPS cells have emerged as a powerful tool to study the G × E with regard to DDs (Schmidt, 2021). Human brain organoids generated from iPS cells with the knockout of chromodomain helicase DNA binding protein 8 (*CHD8*), a strong candidate gene associated with ASD, showed increased susceptibility to chlorpyriphos, an organophosphate pesticide that has adverse effects on the developing nervous system, compared to those from the wild-type iPS cells (Modafferi et al., 2021). Human iPS cell-derived cerebral organoids have also been successfully used to analyze the developmental neurotoxicity of alcohol at the genetic, metabolic, subcellular, cellular, and tissue levels (Arzua et al., 2020). These studies suggest that human brain organoids can be used as versatile models to analyze the G × E associated with DDs.

Public databases such as the Comparative Toxicogenomic Database (CTD) (Davis et al., 2021), Gene Expression Omnibus (GEO) (Clough and Barrett, 2016), Simons Simplex Collection (SSC) (Fischbach and Lord, 2010), and Autism Sequencing Consortium (ASC) (Buxbaum et al., 2012) can be used to discover novel interactions between chemicals and genes associated with DDs. For example, an integrative analysis using CTD, SSC, and ASC revealed a total of 212 gene–environment interaction pairs putatively relevant for ASD, and provided a list of candidate genes susceptible to chemicals associated with ASD, such as valproic acid, benzo(a)pyrene, bisphenol A, particulate matter, and perfluorooctane sulfonic acid (Santos et al., 2019). A novel *in silico* approach using GEO identified tumor suppressors: p53, retinoblastoma 1, and Krüppel-like factor 8 as leading nodes in the network of developmental neurotoxicity of selective serotonin reuptake inhibitors and antidepressants associated with ASD (Li et al., 2021). A study using CTD and a database of ASD gene networks (Nelson et al., 2012) found that ASD-associated genes are selectively targeted by environmental pollutants such as pesticides, heavy metals, and phthalates (Carter and Blizard, 2016). Novel disease-associated genes can be identified using whole exome sequencing of biological samples from patients with DDs (Kuroda et al., 2019a; Kuroda et al., 2019b). CTD can be used to examine whether the novel genes are targeted by environmental chemicals and thereby confirm the role of these genes in the susceptibility to the chemicals (Davis et al., 2021). A database named Human Tissue-specific Exposure Atlas (TExAs) has been developed by compilation of various databases, including CTD, Exposome-Explorer (Neveu et al., 2020), PubChem (Kim et al., 2021), ToxCast (Dix et al., 2007), and DisGeNET (Piñero et al., 2019). Using TExAs, one can retrieve the information about tissue-specific target genes of the chemicals and diseases associated with these genes (Ravichandran et al., 2021). The integration of databases combined with new approach methodologies may provide novel insights into the G × E related to DDs (Li et al., 2019b; Cheroni et al., 2020).
